# A log linear Poisson autoregressive model to understand COVID-19 dynamics in Saudi Arabia

**DOI:** 10.1186/s43088-022-00295-z

**Published:** 2022-09-23

**Authors:** Salem Mubarak Alzahrani

**Affiliations:** Faculty of Arts and Science in Almandaq, Al-Baha University, Al-Baha, Saudi Arabia

**Keywords:** COVID-19, Count time series, Log-linear Poisson autoregressive model, ARIMA model

## Abstract

**Background:**

On March 2, 2020, the first case of COVID-19 infection in Saudi Arabia was identified and announced by the health authorities. From first week of March, the number of new confirmed COVID-cases has gradually increased, reaching 2932 confirmed cases on April 9, 2020. A period of increasing infection cases was noticed in June and July 2020. Many methods have been taken to model and predict the new confirmed cases of COVID-19, such as the traditional time series forecasting method and other several methods.

**Results:**

We present two statistical models, namely the log linear autoregressive Poisson model and the ARIMA model. The COVID-19 infectious dynamics were evaluated using models in Saudi Arabia, which can affect health, economics, finance, and other fields. We applied both models to daily confirmed cases of COVID-19 count time series data. Moreover, we compare the log linear Poisson autoregressive model with the automatic ARIMA model.

**Conclusions:**

The result of this study showed that a log linear Poisson Autoregressive model gives better forecasting and the predicted results of the log linear Poisson Autoregressive model can be used as the baseline for additional interference to avoid future COVID-19 pandemic incidents. Moreover, the application of a log linear Poisson Autoregressive can be comprehensive to other cases in Saudi Arabia.

## Background

Saudi Arabia, like most countries around the world, is under the influence of the effects of the coronavirus pandemic that entered the country on March 3, 2020. COVID-19 is a contagious viral disease that is spread through the breath directly. While coughing or sneezing, the patient, directly or indirectly, by contact, touches the surfaces containing the virus. According to the WHO 2020 report, the coronavirus finds its way into the human body through the mucous layers of the mouth and nose. There were many researchers have recently who proposed mathematical models, statistical model and machine learning algorithms which deals with analysis of factors cause diseases and forecast the future situation of the disease and determine its effects. Ahmed et al. [1], Tian et al. [2], Konstantinos et al. [3], and Agosto et al. [4] have considered a log-linear model for count time series; they studied its probabilistic assets and maximum likelihood estimation and showed that a nervous version of the process is geometrically ergodic. Furthermore, in some circumstances, it was established that the vector of unknown parameters' maximum likelihood estimator is asymptotically average, with a correlation matrix that can be regularly estimated. Their findings are based on the most basic assumptions and can be extended to the issue of a log-linear extraction with various ongoing factors. Agosto et al. [4] presented a statistical model, namely a Poisson autoregression, employed to understand infection dynamics concerning COVID-19, which appears to have a significant influence on health, finance, and economics. The model reveals whether infection has a tendency and where any state stands in relation to that tendency. Their statistics of the reported data for China, Iran, Italy, and South Korea are reported. Ahmed et al. [1] used the math frame paradigm to investigate the role of behavior change in slowing the prevalence of COVID-19 in Saudi Arabia. They used the susceptible-exposed-infection retriever (SEIR). They indicated that social distance, health situations, and travel restrictions are strict measures to stop the prevalence of the COVID-19 outbreak. To analyze and forecast COVID-19 daily new confirmed cases in Saudi Arabia, several models for constructing time series data have been proposed. However, the suitability of any of these models to a given time series data must be judged based on its fit to that data.

In this study, the ARIMA model and a log linear Poison Autoregressive model will be applied to the data of COVID-19, which represents the daily new confirmed Saudi Arabian cases. These models are used to predict and carry out the estimation of parameters, which helps us interpret the indicators on confirm Saudi Arabian cases. The data were updated in real time from March 2020 to January 2022. The Saudi Arabian Ministry of Health collected the data to provide a dynamic epidemiological profile of Saudi Arabia.

This study is organized as follows: Sect. 2 explains the dataset and methods, namely a log-linear Poisson Autoregressive model and an ARIMA model. Section 3 describes the empirical results. Discussions and conclusions are presented in Sects. 4 and 5.

## Methods

In this study, we used an ARIMA model and a log linear Poisson Autoregressive model to forecast and estimate daily confirmed coronavirus cases in Saudi Arabia. Studying of infectious illnesses, count-time series linked to incidence, such as the daily incidence of an infectious disease, are common. This count time series data can be modeled and forecasted using a variety of methods, including deterministic models like the SIR and SEIR models, as well as stochastic models like discrete and continuous time Markov chains and stochastic differential equations.

### The log linear Poisson autoregressive model

To model the daily cases of COVID-19 in Saudi Arabia, which is a countable variable, a Poisson autoregressive is represented as a function of both short-term dependence and long-term dependence for count time series (see [4–7]). Following [8], the number of new confirmed cases $y_{t}$, reported at time *t* (day), is assumed to follow a Poisson distribution, i.e.

$$y_{t} \sim {\text{Poissn}}\left( { \lambda_{t} } \right)$$

As pursued with a log-linear autoregressive density specification

1 $$\log \left( { \lambda_{t} } \right) = \alpha + \beta \log \left( {1 + y_{t - 1} } \right) + \gamma \log \left( { \lambda_{t - 1} } \right),$$

where $\alpha \in R$ is the intercept, $\beta \in R$ is the short-term dependence of the anticipated percentage of case related to time *t*, $\lambda_{t}$ represent all past counts of the observed process. Note that, $\lambda_{t - 1}$ the observed of the previous day (time *t* − 1) and $\log \left( {1 + y_{t - 1} } \right)$ is included rather than $\log \left( {y_{t - 1} } \right)$, to make it possible to deal with the issue produced by null values. The term $\gamma \in R$ relates to a trend component and represents the long-term dependence of $\lambda_{t}$. Negative dependence is possible using a log-linear autoregressive density description rather than a linear one.

### ARIMA models

According to Box and Jenkins [9], an ARIMA $\left( {p,d,q} \right) \times (P,D,Q)^{s}$ model can be written as:

$$\varphi \left( {\rm B} \right)\Phi \left( {{\rm B}^{s} } \right)\nabla_{d} \nabla_{s}^{D} {\rm X}_{t} = \theta \left( {\rm B} \right)\Theta \left( {{\rm B}^{s} } \right)e_{t} ,$$

where $\left( {p,d,q} \right) \equiv$ nonseasonal part of the model, $\left( {P,D,Q} \right) \equiv$ seasonal part of the design, and $S$ is the season length see [7]. Additionally, $p, d$ and $q$ stand for the autoregressive order, the non-seasonal differencing degree and the moving average order, respectively, and $P, D$ and $Q$ are the abbreviations for the seasonal autoregressive order, the seasonal differencing degree, and the seasonal moving average order.

### Evaluation criteria

Very widespread accuracy measurement functions are used to assess the performance of each model. These performance functions are:

- MAE stands for mean absolute error. (MAE):

$${\text{MAE}} = \frac{1}{N}\mathop \sum \limits_{i = 1}^{N} \left| {y_{i} - \tilde{y}} \right|$$,

where $y_{i}$ and $\tilde{y}$ are actual and anticipated ratings, respectively.

- (RMSE) stands for root mean square error:

$${\text{RMSE}} = \sqrt {\frac{1}{N}\mathop \sum \limits_{i = 1}^{N} \left( {y_{i} - \tilde{y}} \right)^{2} }$$.

- Mean absolute percentage error (MAPE):
  $${\text{MAPE}} = \frac{1}{N} \frac{{\mathop \sum \nolimits_{i = 1}^{n} \left| {\tilde{y}_{i} - y_{i} } \right|}}{{\tilde{y}_{i} }} \times 100.$$

### The data

The Saudi Ministry of Health provided the data for this study (https://covid19.moh.gov.sa). It represents COVID-19 confirmed Saudi Arabian incidents from March 3, 2020, to June 10, 2021, and it was used in the examination of the study.

## Results

**Fig. 1:**
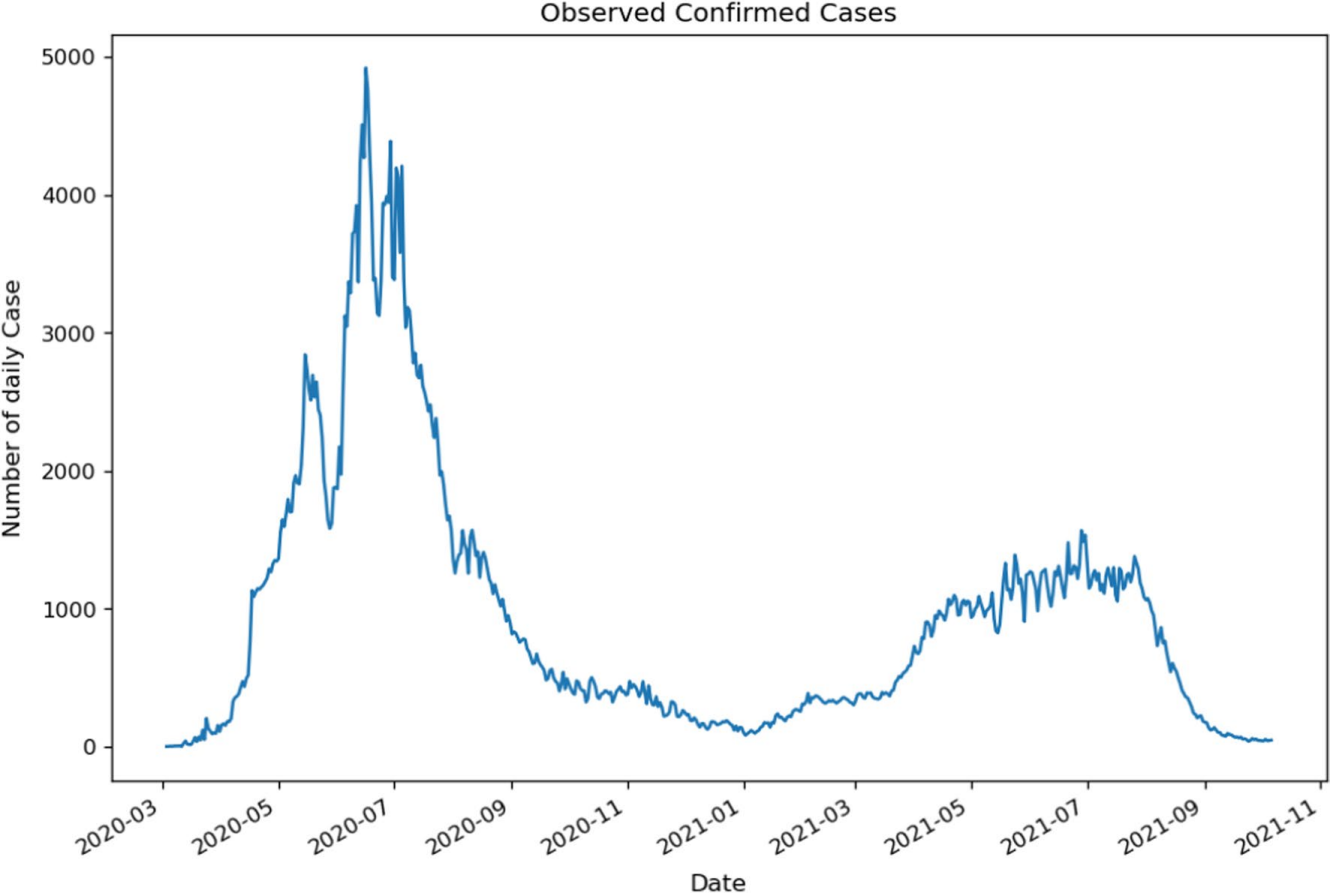
Sequence chart of new series of COVID-19 confirmed cases

This part gives the empirical analysis results on the application of AIM (autoregressive integrated moving average), which is a type of moving average that is used, and A Log Linear Poisson Autoregressive Mode with our data. Figure 1 shows the sequence diagram of daily readings of COVID-2019 confirmed cases during the period from 3/3/2020 to 6/10/2021. COVID-19 confirmed cases show a sharply increasing trend starting in March 2020 up until the close of 2020, and then it decreases slowly until the opening of 2021, between January 2021 and September 2021. The COVID-19 indicates a small growth until August 2021 and a sharp decrease until the completion of the course of study.

### Applying autoregressive linear Poisson model

In this subsection, we have been estimated the parameter *α*, *β* and the log Autoregressive Linear Poisson model for daily confirmed cases of COVID-19 using the language, Count Time Series. jl package, Julia programming [10]. The results are summarized in Table 1.

**Table 1:** The parameter estimates of the log linear Poisson autoregressive model

Parameter	Estimate	*t* statistic	*p* value
$\alpha$	0.77112	12.524	0.031
$\beta$	− 0.107176	24.379	0.019
$\gamma$	0.999994	25.088	0.014

Table 1 illustrates that all estimated autoregressive coefficients are significant, which verifies the presence of both short-term and also long-term reliance on daily cases of past infection counts. Besides that, the sign, which quantifies the long-term part of the Poisson Autoregressive, is positive, thus suggesting the existence of an increasing trend in the expected new daily cases. The estimates for the parameters a negative trend and show a positive trend.

### Applying ARIMA models

This section provides the model specification, estimation, and diagnostic checking data from the COVID-19 instances that have been confirmed during the period 3/3/2020–6/10/21. Augmented Dickey Fuller tests were applied to daily cases of COVID-19 during the period from 3/3/2020–6/10/2021 to assess if the series was stationary or not. From Table 2, we can observe that the series of daily confirmed cases is considered non-stationary.

**Table 2:** ADF test results

Variable	ADF test	
	Level	Prob
COVID-19 confirmed cases	− 5.7615	0.379

**Fig. 2:**
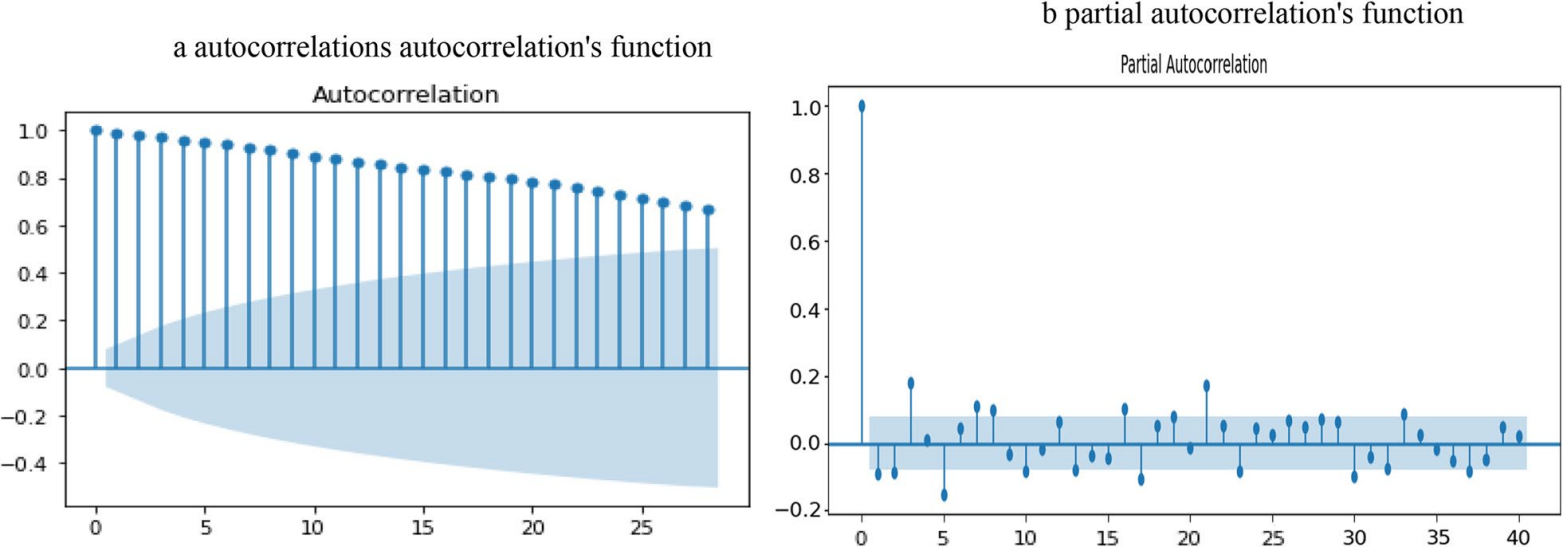
(**a**) autocorrelations autocorrelation’s function. (**b**) Partial autocorrelation’s function

The correlogram of autocorrelations (ACF) and partial autocorrelations (PAFC) of daily confirmed cases of COVID-19 are shown in Fig. 2a,b. It can be seen that in both figures the ACF starts with large positive significant patterns and decays gradually as an increasing lag and also shows a sharp increase in ACF values, while the PAFS shows a large positive peak at lag 1. This result confirms that the COVID-19 confirmed case series is non-stationary as well as an autoregressive model is adequate in modeling and forecasting their future values.

Python code the automatic auto Arima function was applied to the data. The empirical findings revealed that ARIMA (4, 1, and 5) was selected as an appropriate model to represent the data.

## Discussion

Through this work, we have modeled the daily confirmed cases of coronavirus in Saudi Arabia. Using two different models, the first model is a log linear Poisson autoregressive model that offers the ultimate description of the data in terms of both short-term and long-term dependence and the second model is ARIMA model.

Table 3 shows our assessment of the predictive accuracy of our two models. We fitted both models using confirmed cases for the period from 31-10-2021 to 29-11-2021 to get predictions. The predictions are then compared with out-of-sample predictive performance. The study found that a log-liner Poisson Autoregressive model always outperforms the ARIMA model.

**Table 3:** Evaluation criteria’s

Model	MAE	RMSE	MAPE
A log liner Poisson autoregressive	5210.21	72.10	38.02
ARIMA (4,1,5)	6012.12	75.32	39.49

The majority of studies in Saudi Arabia dealt with modeling time series data of COVID-19 using only ARIMA models. It is noted that, applications of ARIMA models ignore the fact that pandemic evolution data is counted. To address this problem, a new combination of a log linear autoregressive Poisson model which dealt with counting in time series data is presented in this paper. As well as comparing the efficiency of the ARIMA and log linear autoregressive Poisson models to select the most suited model for the nature of Saudi data. We found that a log linear autoregressive Poisson model is more accurse than ARIMA model.

### Prediction of new cases of COVID-19

**Fig. 3:**
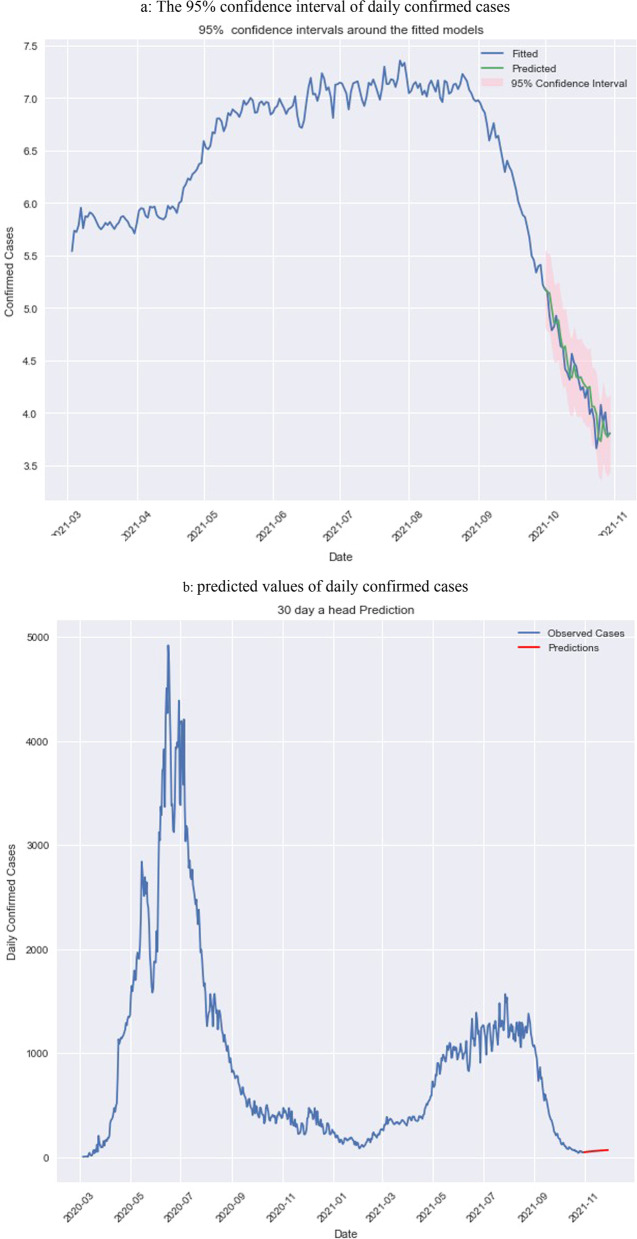
(**a)** The 95% confidence interval of daily confirmed cases. (**b**) Predicted values of daily confirmed cases

The researcher applied a Log Liner Poisson Autoregressive model for prediction for the next 30 days for the period 31-10-2021 to 29-11-2021. Figure 3a shows that all fitted values are within the 95 percent confidence interval, indicating that the model is accurate in predicting future infected cases in Saudi Arabia. While Fig. 3b represents a 95% confidence level in the predicted values.

## Conclusions

In this study, the researcher applied two different models: a log linear Poisson autoregressive model and a traditional ARIMA model. The log-line Short-term and long-term dependencies influence the Poisson autoregressive model that provides the most appropriate for the data. A traditional ARIMA model gives constant prediction. However, the use of the log linear Poisson autoregressive steep paradigm permits the grasping of short-term and long-term memory influences, which can significantly contribute to the development of an assessment of the number of short-term and long-term confirmed new cases. It can show whether a disease has an ascending or descending tendency, which can benefit the common resolution makers for health and policy interventions and suitable measures to stop the prevalence of the virus. The results of this study showed that the log linear Poisson Autoregressive model gives better prediction results. Moreover, the log linear Poisson Autoregressive predicting results might be utilized as the baseline for additional interference to avoid future coronavirus pandemic incidents. Therefore, it is necessary to extend the application of log linear Poisson Autoregressive to other cases in Saudi and improve methods so that they are more appropriate to overcome the estimation problems such as underestimation or merging in long-term forecasting.

## Data Availability

The data used in this study are obtained from ministry of health of Saudi Arabia. Daily data representing COVID-19 confirm cases during the period 3/3/2020–6/10/2021 are used in the analysis of this study. All the data generated and analyzed during the study are included in the manuscript.

## Abbreviations

WHO: World Health Organization
COVID-19: Coronavirus 2019
ARIMA: Auto regressive integrated moving average intravenous
MAE: Mean absolute error
